# Cortisol Reactivity to Acute Psychosocial Stress in Physician Burnout

**DOI:** 10.3390/biomedicines12020335

**Published:** 2024-02-01

**Authors:** Claudia Zuccarella-Hackl, Mary Princip, Sarah A. Holzgang, Sinthujan Sivakumar, Alexa Kuenburg, Aju P. Pazhenkottil, Diego Gomez Vieito, Roland von Känel

**Affiliations:** 1 Department of Consultation-Liaison Psychiatry and Psychosomatic Medicine, University Hospital Zurich, University of Zurich, Haldenbachstrasse 16/18, CH-8091 Zurich, Switzerland; mary.princip@usz.ch (M.P.); sarahandrea.holzgang@usz.ch (S.A.H.); sinthujan.sivakumar@usz.ch (S.S.); alexa.kuenburg@usz.ch (A.K.); aju.pazhenkottil@usz.ch (A.P.P.); roland.vonkaenel@usz.ch (R.v.K.); 2 Department of Cardiology, University Hospital Zurich, University of Zurich, CH-8091 Zurich, Switzerland; 3 Cardiac Imaging, Department of Nuclear Medicine, University Hospital Zurich, University of Zurich, CH-8091 Zurich, Switzerland; 4 Institute of Molecular Cancer Research, University of Zurich, CH-8091 Zurich, Switzerland

**Keywords:** burnout, physicians, stress reactivity, HPA-axis, serum cortisol, Trier Social Stress Test, TSST, Maslach Burnout Inventory, cardiovascular health

## Abstract

Background: Physician burnout, characterized by chronic job-related stress leading to emotional exhaustion, depersonalization, and reduced personal accomplishment. This cross-sectional study investigates cortisol reactivity in male physicians with burnout compared to healthy controls during an acute psychosocial stress test. Methods: Sixty male physicians (30 burnout, 30 healthy controls) participated between September 2019 and December 2021 to investigate the impact of burnout on cardiovascular health. Salivary cortisol levels were measured before and after a Trier Social Stress Test (TSST). Burnout was assessed with the Maslach Burnout Inventory-Human Services Survey (MBI-HSS). Covariates included age, BMI, and physical activity. Data were analyzed using repeated measures analysis and area under the curve analysis. Results: Male physicians with burnout exhibited significantly greater cortisol reactivity during the TSST, notably post-stress to 15 min post-stress. Emotional exhaustion correlated with reduced cortisol increase from pre-stress and smaller post-stress to 15- and 45-min declines. Discussion: Findings suggest heightened cortisol reactivity in male physicians with burnout, possibly reflecting initial chronic stress stages. This study highlights the necessity for long-term research on cortisol’s influence on cardiovascular health and stress responses across diverse groups. Conclusions: The findings contribute to comprehending physiological responses in burnout-afflicted physicians, emphasizing cortisol reactivity’s pivotal role in stress-related research and its potential health implications, particularly within the burnout context.

## 1. Introduction

Burnout can be defined as a syndrome characterized by feelings of exhaustion (EE), depersonalization (DP), and decreased personal accomplishment (PA), resulting from prolonged exposure to job-related stress [1]. The significance of physician burnout is underscored by prevalence rates ranging from 30–40% for moderate to severe burnout in Switzerland, with a tendency of increasing prevalence [2]. The variability in prevalence estimates also highlights international differences in burnout rates [3]. These include its impact on patient care, where studies have demonstrated connections between physician burnout and suboptimal patient care practices [4,5], a doubling of the risk of medical errors [6,7], and a 17% increase in the likelihood of being involved in a medical malpractice lawsuit [8]. Furthermore, physician burnout has implications for the healthcare system, as it has been linked to decreased productivity [9], job dissatisfaction [10], and a more than twofold increase in self-reported intentions to leave one’s current practice for reasons other than retirement [11,12]. Antecedent factors leading to burnout in physicians can be explored on different levels, considering demand-resource gaps with regard to occupational, organizational, social-psychological and intra-individual factors [13]. Finally, physician burnout affects both psychological well-being [14,15] and physical health [5,16], particularly in relation to coronary heart disease (CHD) [17,18].

One pathway through which burnout symptoms may contribute to an elevated risk of CHD involves their potential impact on responses to acute stress situations [19]. The endocrine system reacts to acute stress by triggering the activation of the hypothalamic-pituitary-adrenal (HPA) axis, leading to the subsequent release of cortisol from the adrenal cortex [20]. Cortisol attaches to glucocorticoid receptors in target cells and holds considerable importance owing to its metabolic attributes, offering prompt energy to the organism to manage acute stress scenarios and eventually reestablish homeostasis [21]. Common symptoms such as fatigue, exhaustion, depressed mood, or compromised cognitive function, which are linked with an imbalanced HPA axis, also constitute pivotal manifestations observed in cases of burnout [22].

However, the findings from previously published studies exploring the potential influence of burnout on reactions to acute psychosocial stressors with regard to the HPA axis, have yielded inconsistent results [23,24,25,26,27] (Supplementary Table S1). Some evidence suggests that burnout might lead to reduced responsiveness in this system [24,25,26]. One study that investigated the cortisol response of individuals with burnout applying the Trier Social Stress Test (TSST) found a reduced cortisol response [26]. However, this reduced cortisol response was only evident in severely affected individuals with burnout, while no distinctions in cortisol response compared to healthy controls were observed in individuals with moderate levels of burnout [26]. In support of these findings, another study employing a virtual-reality version of the TSST, focusing on individuals with an exhaustion disorder who scored high on burnout symptoms, likewise reported reduced cortisol reactivity compared to individuals scoring low on the burnout symptoms [25]. In contrast, another study did not find any significant distinctions in cortisol reactivity to the TSST in burnout patients and a healthy control group. Nonetheless, individuals experiencing burnout displayed higher cortisol levels during the initial hour after waking when compared to their healthy counterparts [23]. Finally, Wekenborg discovered that burnout, depressive symptoms, and hair cortisol levels were associated with reduced cardiovascular reactivity during a TSST, with the timing of this impact varying [27].

It follows from this literature that establishing a firm link between burnout and cortisol patterns during stressful situations is challenging due to limited available studies, variations in cortisol secretion patterns, inconsistencies in the measurement of underlying constructs, and the diversity of occupations [22]. In order to address these research gaps, our study sought to explore potential associations between burnout in physicians and alterations in acute stress responses in salivary cortisol. We involved two clearly distinct groups: one comprised of employed male physicians experiencing burnout without severe depressive symptom level, and a control group consisting of healthy, employed male physicians. Additionally, we aimed to explore both shared and distinctive aspects of cortisol stress responses among different burnout symptoms, including the sum-score, EE, DP, and PA. This investigation extends to assessments conducted both during rest and in reaction to acute stress among individuals experiencing burnout. Notable distinctions in cortisol reflecting compromised cardiovascular health between burnout-afflicted individuals and a control group could unveil the underlying mechanisms contributing to an elevated CHD risk during the early stages of burnout. Given the prevalent occurrence of burnout among physicians, our study outcomes have the potential to guide healthcare professionals in identifying those at an escalated risk of cardiovascular disease. This insight could prompt physicians to implement strategies aimed at mitigating burnout and concurrently addressing cardiovascular risk factors.

## 2. Materials and Methods

### 2.1. Participants and Recruitment

The research was granted approval by the local ethics committee in Zurich (BASEC-Nr. 2018-01974), and all participants provided written consent after being fully informed about the study protocol. Data collection was conducted over two years, from September 2019 to December 2021. We recruited male physicians in Switzerland using various methods, including hospitals, clinics, physician associations, and direct email communication, for a study examining the impact of burnout on cardiovascular health among physicians. Interested physicians were provided with information and study objectives through text messages and flyers and were given the opportunity to contact the study management. A total of 60 individuals were enrolled in the study, with 30 participants in each group: the burnout group and the healthy control group. Concerted efforts were made to recruit healthy controls who matched physicians with burnout in terms of age (±5 years), body mass index (BMI) (±5 kg/m^2^) and family history of early CVD in first degree relatives (men < 55 years, women < 65 years). To assess and allocate participants into their respective groups, a telephone interview was conducted, utilizing the Maslach Burnout Inventory-Human Services Survey (MBI-HSS) [28] and the Patient Health Questionnaire 9 (PHQ-9) [29]. Additional criteria for inclusion and exclusion were also reviewed.

In order to establish the criteria for assigning participants to their respective groups, we relied on a previous systematic review focusing on physician burnout [3]. For the clinical burnout group, a cutoff for emotional exhaustion (EE) ≥ 27 and/or depersonalization (DP) ≥ 10 (with a minimum EE score of ≥20) was used, whereas for the control group, the cutoff was EE < 16 and DP < 7. Additionally, for the burnout group a PHQ-9 score of ≤14 was required, indicating at most moderate depressive symptoms. For the control group, a PHQ-9 score of ≤10 was required, indicating at most mild depressive symptoms [29]. Moreover, individuals were eligible for the burnout group if they had experienced workplace stress and significant exhaustion for a minimum of six months prior to their enrollment in the study [25].

Additional criteria for inclusion in both groups were individuals aged between 28 and 65 years (which corresponds to the official retirement age in Switzerland) and non-smokers for a minimum of 5 years. Exclusion criteria for both groups comprised individuals with a history of clinical depression or burnout, diagnosed heart disease, familial hypercholesterolemia, type I or type II diabetes, known stage II hypertension, renal insufficiency, any active serious disease, use of lipid-lowering, antihypertensive, or antidiabetic medications, BMI ≥ 35 kg/m^2^, chronic risky alcohol consumption, contraindications for adenosine, beta-blockers, or nitrates, allergy to iodine-containing contrast media, medication affecting blood biomarker levels (such as corticosteroids and anticoagulants), and a choice to forego disclosure of clinically relevant cardiac imaging findings.

It should be noted that the two-year data collection period poses a potential source of research performance bias, as information such as the study participant’s status over the entire 2-year period (including the occurrence of diseases during the study and changes in the work environment) is lacking.

### 2.2. Study Procedure

In the Nuclear Medicine Department, cardiac imaging was conducted between 7:10 a.m. and 9:10 a.m. Following that, the participants were brought to the Stress and Behavior Research Lab within the Department of Consultation-Liaison Psychiatry and Psychosomatic Medicine, where they underwent the TSST and additional assessments. At 9:40 a.m., they received a light standardized breakfast consisting of two rolls, an apple, and water. Subsequently, they were equipped with either the Finapres^®^ Nova (Finapres Medical System, Enschende, The Netherlands) or the Omron Evolv device (Omron Healthcare Co., Kyoto, Japan) to monitor heart rate (HR) and blood pressure (BP) during the TSST. However, technical issues with the Finapres^®^ Nova led to only the first 13 participants having their HR and BP readings recorded using this device. HR was further measured with the FAROS 180, a high-End 1 Channel Portable ECG Monitor (Biosystems Ltd., Gronau, Germany). Skilled laboratory staff collected blood samples during the TSST involved the use of an 18-gauge catheter inserted into an antecubital vein, which had been previously utilized for cardiac imaging. During the recovery phase of the TSST, participants were requested to disclose information about their demographics and health behaviors and to fill out psychometric questionnaires. Following the recovery phase, participants received a debriefing that clarified the test’s intention as inducing stress rather than evaluating their personal abilities.

### 2.3. Trier Social Stress Test

The TSST has been applied as a widely used standard protocol to induce acute psychosocial stress and Cortisol responses under laboratory conditions [30]. The TSST combines a short 2-min (min) introduction phase followed by a 3-min preparation phase, a 5-min speech stress (i.e., a mock job interview), and a 5-min mental arithmetic task in front of an audience (trained staff) and a video camera. The specific arrangement of this scenario is widely recognized as highly stressful due to its elements of social evaluation and lack of control. Saliva samples to determine cortisol levels were collected at five-time points starting at 10:00 a.m.: 10 min before the instruction (“pre-stress”), immediately after the TSST (“post-stress”), “15 min post-stress”, “45 min post-stress”, and “90 min post-stress”.

### 2.4. Psychometric Assessment

To evaluate burnout, the 22-item MBI-HSS in German was utilized, comprising three subscales: EE (9 items), DP (5 items), and PA (8 items) [28]. Each item is scored on a scale ranging from 0 (“never”) to 6 (“daily”). EE evaluates the sense of energy depletion and exhaustion from work, DP gauges a detached and cynical attitude toward patients or care recipients, and PA explores feelings of competence and successful job performance as a physician. The subscales can be individually analyzed. In our sample, the internal consistency (Cronbach’s α) was 0.95 for the EE subscale, 0.87 for the DP subscale, and 0.77 for the PA subscale.

We evaluated job stress by using a shortened version of the Effort-Reward Imbalance (ERI) questionnaire in German. The questionnaire consisted of three items related to work effort and seven items regarding the rewards received at work [31]. Each item was rated on a 4-point scale, ranging from 1 (“strongly disagree”) to 4 (“strongly agree”). To calculate the effort-reward ratio, a correction factor was applied to account for the unequal number of effort and reward scores. A higher ratio indicated higher levels of job stress. Cronbach’s α was 0.76 for the effort scale and 0.77 for the reward scale in our sample.

We measured depressive symptoms experienced in the past two weeks using the German version of the PHQ-9 questionnaire [32]. The questionnaire consisted of nine items that participants rated on a 4-point Likert scale, ranging from 0 (“not at all”) to 3 (“nearly every day”). The total score on the scale ranged from 0 to 27, with higher scores indicating a higher severity of depressive symptoms. In our sample, Cronbach’s α for the PHQ-9 total scale was 0.79.

### 2.5. Health Behavior Assessment

The Body Mass Index (BMI) was calculated by dividing the weight measured in kilograms by the square of the height measured in meters. The evaluation of physical activity focused on moderate-intensity or vigorous sports activities. In order to gather this information, participants were inquired with the following question: “On average, how frequently do you engage in sports activities per week that lead to sweating?” Answers ranged from 0 to 7 times.

### 2.6. Biochemical Analyses

Salivary cortisol: To measure cortisol levels, saliva samples were gathered using salivettes (Sarstedt, Rommelsdorf, Germany), preserving them at −80 °C until analysis. Upon thawing, the saliva samples underwent centrifugation at 3000 rpm for 10 min to obtain saliva with low viscosity. Cortisol levels were determined using an enzymatic assay, where standard values were fitted using 4 parameter logistics, in accordance with manufacturer’s instructions. Salivary free cortisol concentrations were expressed in ng/mL. Intra-assay coefficient of variation (CV) was 1.38%, and inter-assay CV was 5.9%.

### 2.7. Data Analysis

Data were analyzed using SPSS 29.0 for Windows (Armonk, NY, USA: IBM Corp). All analyses were two-tailed, with level of significance at *p* < 0.05. To address non-normal distribution, a two-step method was used to transform the values of cortisol levels at each measurement time point into a statistically normal distribution [33]. In the initial step, the continuous variable was converted into a percentile rank, ensuring evenly distributed probabilities. Subsequently, in the second step, the output of the first step underwent an inverse-normal transformation, resulting in a variable composed of z-scores that conform to a normal distribution, while retaining the original series mean and standard deviation [33].

Repeated Measures Analysis of Covariance (RM ANCOVA) was employed with ‘time’ as the within-subject factor and ‘group’ as the between-subject factor to evaluate differences in cortisol activity measures between the burnout and control groups across the four time points under consideration. Covariates utilized in the study were age, BMI, and physical activity. The latter two covariates were selected a priori as they have been shown to be associated with cortisol reactivity to psychosocial stress test [34,35]. Age was controlled for as the burnout group was found to be significantly younger than the control group, despite attempts to match groups on age. To prevent inaccurate coefficient estimates caused by the strong correlation between depressive symptoms, ‘group,’ and the PHQ-9 total score was not included as a covariate due to high multicollinearity. To rectify violations of the assumption of sphericity, the degrees of freedom were modified using the Greenhouse-Geisser correction. If a variable showed significant effects both within and between subjects, post-hoc analyses were conducted to identify noteworthy correlations between that variable and alterations in cortisol activity measures between two time points, as well as cortisol activity measures at individual time points.

The area under the curve (AUC) was determined using a previously described trapezoid formula to quantify the total stress-induced increase (output) in cortisol activity measures over time [31]. This approach takes into account the varying time intervals between the five measurements conducted in our study, including the 15-min interval from the start of the instruction to the end of the TSST, as well as two recovery intervals of 15 and 30 min, respectively. This method does not consider the distance from zero and focuses specifically on the AUC with respect to the increase, representing the area between the stress response curve and a baseline established as the pre-stress level of cortisol activity measurement. Univariate ANCOVA was performed to examine group differences in the AUC of cortisol activity measures, using the same covariates as in the RM ANCOVA. Partial eta squared (ηp^2^) is utilized to represent the effect sizes. Effect sizes of 0.01, 0.06, and 0.14 for eta squared (η^2^) representing small, medium, and large effects, respectively [36]. In the case of cortisol, one participant had a solitary missing value, which was approximated using the mean of the two adjacent sample values. In another participant, a cortisol value fell below the detection limit, and it was substituted with 0 as the lower limit of quantification. One participant lacked MBI and PHQ-9 data on the examination day; instead, we utilized the corresponding data gathered during the telephone interview.

## 3. Results

### 3.1. Sample Characteristics

Table 1 shows the characteristics of the 60 male physicians, 30 with burnout and 30 controls without burnout. Compared to controls, physicians with burnout were significantly younger and had greater job stress and more severe depressive symptoms. Health behaviors were similar in the two groups. Values of blood pressure and heart rate were reported elsewhere [37].

### 3.2. Cortisol Response to Stress

**Figure 1:**
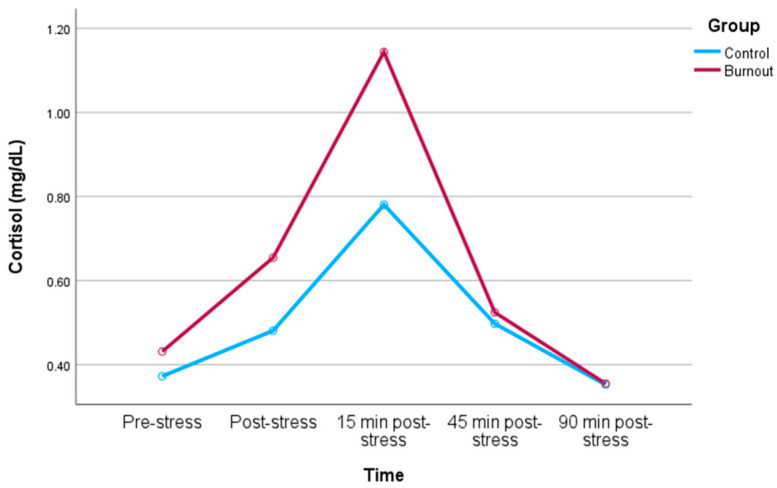
Cortisol reactivity to TSST in burnout physicians and controls.

Repeated measures analysis: The within-subjects analysis depicted a time-by-group interaction for cortisol (F = 4.22, *p* = 0.021; ηp^2^ = 0.068), indicating that the change over time in cortisol levels exhibited a significant difference between participants with and without burnout (depicted in Figure 1). Post-hoc analyses examining changes in cortisol levels between individual time points revealed a more pronounced increase in cortisol from post-stress to 15 min post-stress (F = 4.32, *p* = 0.042, ηp^2^ = 0.069) in the burnout group compared to the control group. This outcome retained significance even after adjusting for covariates (RM ANOVA for time-by-group interaction: F = 3.69, *p* = 0.034; ηp^2^ = 0.063). Within-subjects analysis of individual MBI dimensions indicated an interaction between time and EE (F = 3.40, *p* = 0.025; ηp^2^ = 0.065), but no significant interactions between time and DP (*p* > 0.09) or low PA (*p* = 0.24). Specifically, higher EE was linked to a smaller cortisol increase from pre-stress to post-stress (rp = −0.35, *p* = 0.012) and a smaller cortisol decrease from post-stress to both 15 min (rp = −0.32, *p* = 0.023) and 45 min (rp = −0.34, *p* = 0.016) post-stress. This result persisted even without controlling for any covariates (RM ANOVA for time-by-EE interaction: F = 3.67, *p* = 0.018; ηp^2^ = 0.066). The between-subjects analysis revealed no effects for group and any covariate on cortisol levels (all *p*-values > 0.11).

Area under the curve analysis: No group differences was found with AUC of cortisol (*p* = 0.516, ηp^2^ = 0.007), even after controlling for covariates (*p* = 0.458, ηp^2^ = 0.010). The analysis of individual MBI dimensions showed no associations with total cortisol output (all *p*-values > 0.122).

## 4. Discussion

The aim of this paper was to explore the connection between cortisol stress reactivity in male physicians with burnout, excluding those with severe depressive symptoms, in comparison to a control sample of healthy physicians without burnout. Our findings revealed a significantly greater cortisol reactivity in male physicians with burnout when compared to the healthy controls during the TSST, particularly in terms of an increased cortisol response from post-stress to 15 min post-stress. While the analysis of individual dimensions of the MBI showed no links with total cortisol output, higher EE was linked to a smaller cortisol increase from pre-stress and a smaller cortisol decrease from post-stress to both 15 min and 45 min post-stress.

In contrast to prior research, which discussed either reduced cortisol reactivity in severely affected burnout individuals [25,26], no difference in TSST reactivity between individuals with moderate burnout symptoms and healthy controls [26], or no difference in reactivity [23], our findings intriguingly suggest increased cortisol reactivity in male individuals with burnout. Several explanations for this intriguing finding may apply.

Firstly, at the onset or during extended periods of work-related stress, the HPA axis may respond by becoming hyperactive, leading to increased cortisol levels, and eventually reaching a point of exhaustion, resulting in an overall reduction in cortisol secretion [38]. This aligns with our results, showing that patients with higher EE were associated with a smaller cortisol increase from pre-stress. Consequently, in the study conducted by Penz et al. [39], it can be inferred that many participants interested in participating in a longitudinal burnout study were in the initial phase of chronic work-related stress. A subsequent longitudinal analysis of the same sample further supports this dynamic adaptive model, revealing reduced cortisol levels in individuals with higher work-related stress after a two-year period [39]. This aligns with our study participants, consisting of physicians experiencing burnout who are currently employed. It is reasonable to infer that the likelihood of leaving the job due to burnout will escalate with a prolonged duration of burnout, which could explain their continued heightened cortisol reactivity. Nevertheless, due to our data, we cannot ascertain the duration of the burnout symptoms, which would need to be investigated in future research. Secondly, the results could also be related to the concept of allostatic load, which postulates that repeated adaptation of the body to stressors results in health-related attrition [40,41,42]. Accordingly, the increased signs of stress or allostatic load, here measured by EE, could manifest in a reduced cortisol response, compared to the healthy controls, indicating a dysregulation of the hypothalamic-pituitary axis. Thirdly, another potential factor contributing to the observed findings examining HPA-related measures may be the substantial heterogeneity among individuals (cortisol awakening response (CAR), hair cortisol, serum cortisol), resulting in many inconclusive findings. This mixed set of findings could also be attributed to differences in the occupational groups and genders being studied.

Lastly, our sample consisted in part of physicians, who are (night) shift workers (*n* = 35). In this context, a study with day shift workers showed that global burnout and EE were associated with a higher CAR [43]. In healthy night shift workers, significant changes in urine and serum cortisol, compared to non-night shift workers, were observed, along with a risk for delayed recovery of the circadian rhythm [44], which is linked to cardiovascular health. A study found that a stress test in a laboratory setting led to an increased CAR and cortisol reactivity [45]. Longitudinal studies could investigate whether the hyperactivity of cortisol might shift to hypocortisolism with a longer duration and greater intensity of burnout symptomatology. Since our sample only partially consisted of physicians with shift work, the link between shift work among physicians and their cortisol reactivity could be examined more closely in a homogeneous sample in the future.

Another important aspect is the gender-related effects on salivary cortisol reactivity to the TSST, where higher cortisol values at peak and recovery were observed in men compared to pre-menopausal women [46]. Specifically, the question why men show a greater HPA axis activation and cortisol response to the TSST than do women in the follicular phase of the menstrual cycle has been discussed [47]. Hypercortisolism has also been shown in a sample of burnout patients, with sex-related differences in results. For instance, one study demonstrated that dysregulation in HPA-axis activity, assessed by CAR, was increased in female patients with moderate burnout [48].

Regarding clinical implications, stress reactivity patterns are likely linked to future health outcomes. While blunted stress reactivity is discussed as a predictor for various health issues such as adiposity, obesity, depression, anxiety, PTSD symptoms, and increased illness frequency, exaggerated stress reactivity is discussed as increasing risk factors for cardiovascular disease and decreased telomere length [37].

The strengths of our study include the inclusion of a well-defined group of male physicians with and without burnout, as well as the incorporation of a standardized stress-test. However, it also comes with several noteworthy limitations. Possibly, the results may have been affected by potential biases stemming from the extended two-year data collection period. We examined a sample of male physicians without a history of CVD; consequently, our results do not allow for generalization to other occupational groups, females, or individuals with manifestations of CVD. Considering our moderate sample size, replication in larger samples are needed. The sample size also prevented us from considering additional covariates. BMI and physical activity were selected based on the available literature [49,50]. Especially, due to our study design, it was not possible to consider the specific influence of depressive symptoms, consequently shedding more light on the mixed picture emerging from studies showing the cortisol reactivity to TSST in individuals with MDD [22] remains a future task. Original cortisol measurement time points in the TSST included Baseline (pre-stress), Post-Stress (immediately after), and Post-Acute Phase (minutes and hours after). Omitting the 30 and 60 min post-stress is for practical reasons, considering previous research suggesting their lesser informativeness and relevance.

Cortisol collection can be carried out through various methods such as saliva, blood, urine, and hair, each with the potential to yield different outcomes. However, saliva cortisol collection is less invasive, reducing participant burden and stress. It’s a practical option that doesn’t require specialized training. Salivary cortisol aligns with short-term stress responses crucial for the TSST, while hair cortisol indicates long-term stress. Urinary cortisol reflects an integration of levels over time, providing insights into overall cortisol output and chronic stress. Salivary cortisol specifically reflects biologically active cortisol, distinguishing it from blood measurements that include both free and protein-bound cortisol. Future work should consider measuring both hair cortisol and saliva cortisol to gain insights into physicians experiencing chronic stress and eliciting stress reactions.

Moreover, future research should emphasize the importance of studying cortisol response, in order to better understand what stress-related factors mediate future health consequences. This could establish more individualized stress reactivity patterns in different subgroups like larger samples with women and patients with comorbidities. Future studies should also examine the relationship between depression and burnout more closely in order to achieve a better separation between the two disorders and to show the different effects on cortisol reactivity. The methodology for cortisol collection (e.g., blood, urine, hair) may also have an impact on the results, so different samples should be considered. For example, in general the correlation between cortisol in serum and saliva is shown to be high [51]. In situations with high cortisol in serum, such as after a stress test/stressful situation, on the other hand, the correlation between serum cortisol and saliva cortisol is lower [52].

## 5. Conclusions

In conclusion, this research identified a greater cortisol reactivity in clinical burnout under acute psychosocial stress conditions in male physicians. To confirm the potential impact of this mechanism on cardiovascular health over time, longitudinal studies are needed, aligning with the allostatic load concept. Screening burnout in physicians, along with guidelines for stress management techniques, regular physical activity, a healthy diet, and sleep management to support HPA function, should be developed.

## Figures and Tables

**Table 1:** Characteristics of the 60 study participants.

Variable	Burnout Group (*n* = 30)	Control Group (*n* = 30)	*p*-Value
Age, years	46.77 (10.56)	52.93 (7.48)	0.012
Body mass index, kg/m^2^	25.63 (3.08)	24.35 (2.72)	0.095
Exercise, times/week	1.99 (1.62)	2.67 (1.92)	0.147
Emotional exhaustion, score	29.17 (7.13)	6.67 (3.99)	<0.001
Depersonalization, score	11.33 (7.00)	3.07 (3.60)	<0.001
Personal accomplishments, score	12.03 (6.74)	5.67 (4.37)	<0.001
Effort, score	10.65 (1.36)	8.07 (2.13)	<0.001
Reward, score	19.58 (4.03)	22.24 (2.96)	0.005
Effort-Reward ratio	1.34 (0.41)	0.87 (0.27)	<0.001
Patient Health Questionnaire-9, score	7.40 (3.13)	2.20 (1.97)	<0.001
Shift workers	18	17	1.000

The reported values represent the mean and standard deviation (in parentheses). Independent samples *t*-test was utilized to compare the means of the variables between the burnout and control groups.

## Data Availability

Data are contained within the article.

## Supplementary Materials

The following supporting information can be downloaded at: https://www.mdpi.com/article/10.3390/biomedicines12020335/s1, Table S1: Study Overview.
