# Supplementary material for: Cortisol Reactivity to Acute Psychosocial Stress in Physician Burnout
Source: Biomedicines. 2024 Feb 1;12(2):335. doi: 10.3390/biomedicines12020335 (PMC10886584; doi:10.3390/biomedicines12020335)
Supplement: Supplementary file 1 [file biomedicines-12-00335-s001.zip › biomedicines-2682185-supplementary.pdf]

Supplements

Table S1. Study Overview.

Authors and Year of Publication	Sample (Number and Characteristics)	Burnout Operationalization	Stress Test	Cortisol-Biomarkers	Measurement Time	Controlled Variables	Results
De Vente et al., 2015	Burnout: 55 (M=34, F=21)<br>Healthy Controls: 40 (M=16, F=24)	Maslach Burnout Inventory-General Survey (MBI-GS)	acute psychosocial (speech preparation task, mental arithmetic task, and speech task)	Saliva cortisol	-4min, +5min, +20min, +35min, +50min	Effect modification and stratification for gender; age and BMI as covariates; additionally menstrual phase, oral contraceptive use and menopausal status as covariates for women.	In Male patients, cortisol reactivity was lower than in healthy males. In female patients a tendency for lower basal cortisol was found compared to healthy females.
De Vente et al., 2003	Burnout: 23<br>Healthy Controls: 23	Maslach Burnout Inventory-General Survey (MBI-GS)	adapted version of the Trier Social Stress Test (TSST)	Saliva cortisol	Laboratory session: -4min, +5min, +19min, +33min, +47min	Age and gender as covariates	Basal cortisol levels, cortisol reactivity were similar for burnout patients and healthy controls. Burnout

					Morning cortisol level on another day: awakening time, 30 and 60mins after awakening, and at 12:00		patients showed higher cortisol levels during the first hour after awakening in comparison to healthy controls.
Jönsson et al., 2015	Group 1: former patients recovered from work-stress-related exhaustion (n=14)<br><br>Group 2: participants in pre-stage of exhaustion disorder (experiencing work-related stress in the past six months, but no contact with the healthcare system) (n=17)	Shirom–Melamed Burnout Questionnaire (SMBQ)	Virtual reality version of TSST (V-TSST), twice within one week	Saliva cortisol	Twice before the V-TSST (baseline and preparation phase); initially after V-TSST; +10, +20, +30, +40min after V-TSST	Age, gender and the three subscales of Symptom Checklist-90 (SCL-90 – Somatization, Depression and Anxiety) as covariates	Cortisol increased during the V-TSST and decreased afterwards during the recovery phase. The cortisol was lower during the second session on another day.<br>Lower SMBQ ratings were associated with stronger cortisol responses than with higher SMBQ ratings.

	Group3: Control Group, no experience of work stress in the past six months (n=20)						In Men the cortisol response to the V-TSST was higher than in women, men generally had higher cortisol levels.
Lennartsson et al., 2015	19 patients (9 men, 10 women); 37 healthy subjects (20 men, 17 women)	Shirom-Melamed Burnout Questionnaire (SMBQ)	Trier Social Stress Test (TSST)	Blood samples and saliva cortisol	Blood sample: -10, 0, +20, +30, +40, +50min<br><br>Saliva sample: -10, 0, +10, +20, +30, +40, +50, +60min	-	In patients and healthy controls higher levels of cortisol after the TSST were observed. Patients with higher burnout scores had lower saliva cortisol responses than controls.
Wekenborg et al., 2019	71 employed male participants with varying severity of burnout symptoms randomized in to	German Version of the Maslach Burnout Inventory-	Trier Social Stress Test for Groups (TSST-G)	Hair cortisol and saliva cortisol	-40min, -1min, +12min, +25min, +35min, +45min, +60min, +85min	Controlled for age	Burnout and hair cortisol levels were associated with reduced cardiovascular

	stress condition (n=35) and control condition (n=35)	General Survey (MBI-GS)					reactivity, with the timing of this impact varying.
Penz et al., 2019	N = 150, participants living near the city of Dresden in Germany, data from the prospective cohort Dresden Burnout Study	Work stress, Effort-Reward Imbalance (ERI)	-	Hair cortisol	Once at baseline and follow up	Sex, age, body mass index (BMI), medication, time interval (between baseline and follow up)	Reduced cortisol levels in individuals with higher work-related stress after a two-year period.
Marchand et al., 2014	Day shift workers (N=401), in 34 diverse Canadian workplaces	Maslach Burnout Inventory 16-item General Survey (MBIGS-16)	-	Saliva Samples	5 samples a day: at awakening, 30min after awakening, 14:00, 16:00, bedtime; for 3 days (one rest day, 2 working days) over the course of a week	Adjusted for self-reported time of awakening, sex, age, season of sampling, cigarette smoking, alcohol consumption, physical activity, psychotropic drug use, physical health problems, and	Global burnout and feelings of exhaustion were associated with a higher cortisol awakeing response (CAR) in day shift workers.

						body mass index (BMI)	
Lim et al., 2020	325 shift fire fighters in Korea	Fatigue Severity Scale (FSS)	-	Urinary and serum cortisol	During day, night and every 24h per shift cycle	Gender, age, chronotype, depression, job, post traumatic stress disorder, sleep disorder, fatigue, caffeine intake, subjective health condition, and sleep quality	Significant changes in urine and serum cortisol in healthy night shift workers compared to non-night shift workers, along with a risk for delayed recovery of the circadian rhythm.
Dienes et al., 2019	54 female participants, undergraduates (aged 17 to 23)	Life Stress Interview (LSI)	Trier Social Stress Test (TSST)	Salivary cortisol	Five weekdays at waking and 30min past waking for cortisol awakening response (CAR); baseline, post TSST, after +10min, +25min and +40min after TSST	Wake time, sleep aggregate (e.g. sleep quality), age, psychological distress aggregate (e.g. depression, anxiety, early adversity severity), menstrual cycle, medication, electronic	Stress test in a laboratory setting was associated with an increased cortisol awakening response (CAR) and cortisol reactivity.

						medication electronic monitoring system (MEMS)	
Grossi et al., 2005	Three groups with varying levels of burnout scores:<br><br>Group 1: Twenty-two patients on sick leave due to burnout<br><br>Group 2: Twenty-two working participants with low burnout<br><br>Group 3: 20 working participants with moderate burnout	Shirom Melamed Burnout Questionnaire (SMBQ)	-	Salivary cortisol	At awakening, +15min, +30min, +60min after awakening	Antidepressant medication, time of awakening, sleep variables, negative mood	Dysregulation in HPA-axis activity, assessed by cortisol awakening response (CAR), was increased in female patients with moderate burnout.
